# Management of post-cervical laminectomy fusion pain syndrome with a successful trial of spinal cord stimulation

**DOI:** 10.1097/PR9.0000000000000981

**Published:** 2021-12-21

**Authors:** Layth Dahbour, Thelma B. Wright, Laert Rusha, Pushpinder Uppal, Kanchana Gattu, Seung J. Lee, Blake Watterworth, Lynn Stansbury

**Affiliations:** a Department of Anesthesiology, University of Maryland Medical Center, Baltimore, MD, USA; b Shock Trauma Associates, University of Maryland Medical Center, Baltimore, MD, USA

**Keywords:** Neuromodulation, Cervicalgia, Epidural fibrosis, Case report, Failed-back surgery syndrome, Neuropathic pain, Spinal cord stimulator

## Abstract

**Introduction:**

Spinal cord stimulation has been shown to be beneficial in various postsurgical neuropathic pain syndromes, but the already small cervical epidural space due to epidural fibrosis makes cervical spinal cord stimulator placement very difficult. We present a case of successful cervical cord stimulator implantation in a patient with a history of anterior cervical discectomy and fusion, posterior cervical fusion, and significant epidural fibrosis.

**Methods:**

A 48-year-old woman with a history of type 2 diabetes, nonalcoholic steatohepatitis, and fibromyalgia presented with trauma-induced cervicalgia and bilateral upper extremity radiculopathy.

**Results:**

In a 4-day trial of stimulation, she reported an 80% reduction of her pain and significant improvement in her quality of life.

**Discussion:**

Although anecdotal evidence and case series have shown spinal cord stimulation to be successful in cervical failed back surgery syndrome, we are the first to discuss the technical challenges and complications associated with epidural fibrosis.

## Abstract 2

The cervical epidural space due to fibrosis makes cervical spinal cord stimulator placement difficult. We present a successful case and discuss the technical challenges associated with this frequently encountered pathophysiology.

## 1. Introduction

Epidural fibrosis after spinal surgery is common, reportedly as high as 95% to 100%.^4^ Nerve fibers wrapped in scar tissue are subject to vascular compromise, increased tension, and impaired axoplasmic transport, all believed to contribute to failed back surgery syndrome and complex regional pain syndrome.

Spinal cord stimulation, the most commonly used implantable neurostimulation modality for the management of pain, has been shown to be beneficial in the treatment of the pain syndromes noted above.^8,11^ However, the already small cervical epidural space with additional postsurgical epidural fibrosis can make cervical spinal cord stimulator placement very difficult.

We present a case of successful cervical spinal cord stimulator implantation in a patient with a history of anterior cervical discectomy and fusion, posterior cervical fusion, and significant epidural fibrosis.

The patient provided HIPAA-compliant written consent for their clinical information to be included for publication in this case report.

## 2. Methods

### 2.1. Patient presentation

**Figure 1.**
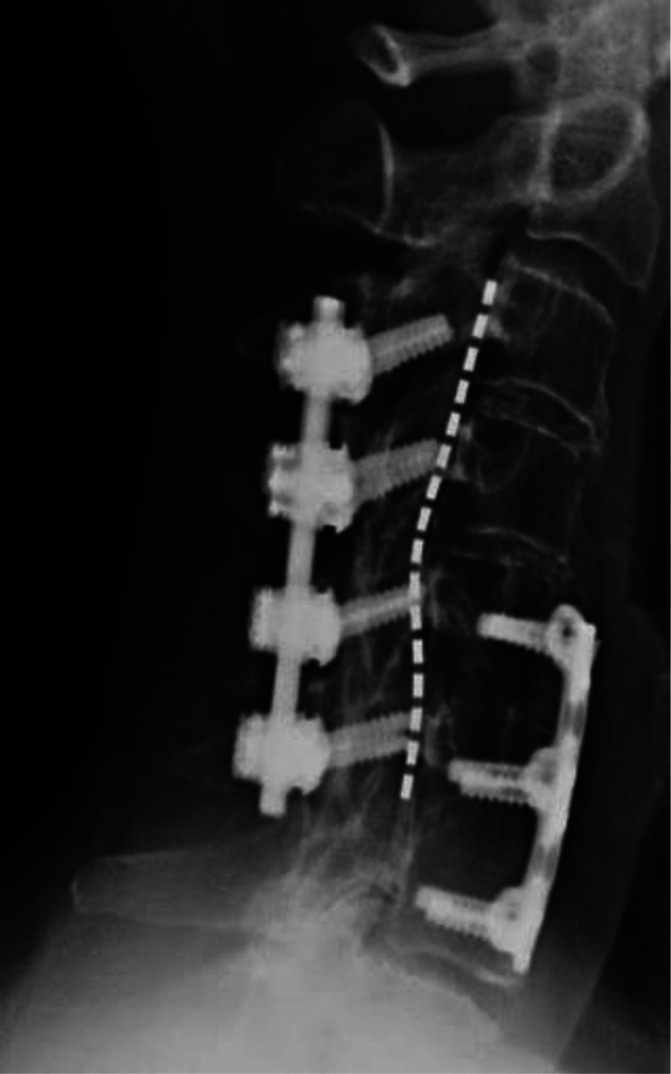
Lateral view x-ray of patient's cervical spine obtained through fluoroscopy. Spinal cord stimulator lead shown to be placed at the bottom of C1 vertebral body with anterior and posterior hardware present.

A 48-year-old female optometrist with a history of type 2 diabetes, nonalcoholic steatohepatitis, and fibromyalgia, presented with trauma-induced cervicalgia and bilateral upper extremity radiculopathy exacerbated by the ergonomic demands of her profession. She had undergone C5 to C7 anterior cervical disk fusion in 1991, C3 to C6 posterior cervical decompression and fusion in 2006, and cervical rhizotomy in 2009 (Fig. 1).

In 2012, she presented to the University of Maryland Pain Clinic with continued severe (8/10) neck and bilateral upper extremity radiculopathy. Over several years, her pain was well-controlled with cervical epidurals, trigger point injections, and facet medial branch blocks. She was maintained pharmacologically on pregabalin (Lyrica, Pfizer), duloxetine (Cymbalta, Lilly), and cyclobenzaprine, with break-through oxycodone 5 mg twice a day. However, in 2017, she noted increased right upper extremity pain in a nondermatomal distribution with vasomotor and sudomotor changes, consistent with complex regional pain syndrome, and required repeat stellate ganglion blocks for pain control. Given multiple cervical surgeries in the past, a compromised and fibrotic epidural space on magnetic resonance imaging, and her history of fibromyalgia, she was not a good candidate for neuromodulation to this area. However, with failure to respond to conservative therapy, the need for break-through oxycodone, frequent clinic visits, and repeated procedures with minimal relief, the careful decision to proceed with a spinal cord stimulation trial was made. The patient underwent psychological evaluation by our pain psychologist before the procedure and subsequently provided written consent.

### 2.2. Procedure

**Figure 2.**
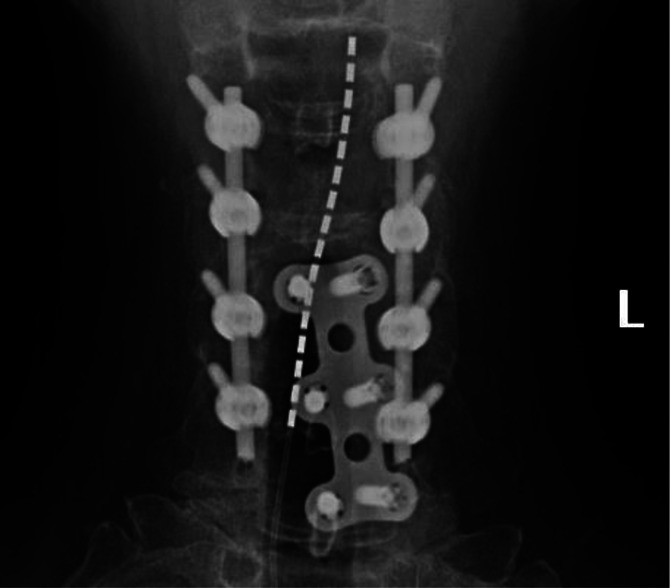
Anteroposterior view x-ray of patient's cervical spine obtained through fluoroscopy. Spinal cord stimulator lead shown to be placed at the bottom of C1 vertebral body with anterior and posterior hardware present.

In our outpatient procedure center under sterile conditions, a Tuohy needle was inserted at T1-2 level to avoid hardware and fibrotic tissue. Once the needle tip was confirmed in the epidural space by fluoroscopy and loss of resistance, a single 16-contact lead (Boston Scientific, Marlborough, MA) was advanced paramedian into the epidural space with minimal difficulty despite the radiographic evidence of epidural fibrosis. Tonic stimulation was used, and the patient reported relief once the lead was placed at the bottom of the C1 vertebral body (Figs. 1 and 2).

## 3. Results

In a 4-day trial of stimulation, our patient reported 80% pain reduction and significant improvement in quality of life. She also reported the elimination of break-through oxycodone from her medication regimen. She would like to pursue permanent spinal cord stimulation placement, but at present, her uncontrolled diabetes (hemoglobin A1c routinely 12%) precludes her from permanent implants. The patient was encouraged to improve the management of her diabetes with her primary care physician and endocrinologist before permanent placement. Notably, the patient and providers acknowledged the possible barrier to insurance coverage, a common obstacle for patients receiving permanent implantation.

## 4. Discussion

This report details successful outcome of spinal cord stimulation for a cervical failed back syndrome associated with epidural fibrosis. Adhesions and/or epidural fibrosis are very common after spinal surgery,^4,5^ do not appear to diminish over time,^14^ and can make lead placement access exceptionally challenging. We believe we are the first to present a successful spinal cord stimulation trial in this situation. As noted in the case presentation, we were pleasantly surprised by the relative ease of lead placement. We continue to monitor this patient closely and as yet have seen no complications.

According to a recent meta-analysis, current evidence for the use of spinal cord stimulation in failed back surgery syndrome carries a positive recommendation with 1B evidence (one or more randomized controlled trials where benefits clearly outweigh risks),^12^ and anecdotal evidence and case series support the use of cervical spinal nerve stimulation in cervical failed back surgery syndrome.^10^ This compares favorably with conservative medical management and with repeat surgery for pain relief, functional status, quality of life, medication utilization, and patient satisfaction. Data are less robust for locally invasive procedures for refractory pain, although anecdotal reports and several series have suggested similar efficacy.^3,7,10,13^ Denis et al have published their experience with the use of laminectomy in cervical lead placement.^6^

The procedure is not without risk. Cervical spinal cord stimulator lead placement itself has been reported to lead to epidural fibrosis,^2^ so a high degree of suspicion for complication from fibrosis must be maintained through follow-up. The patient whose experience is reported here continues to be seen every 4 weeks for reassessment. The use of spinal cord stimulation has been associated with adverse events as high as 30% to 40%, including physiologic microenvironmental and macroenvironmental changes: neurologic injury, lead migration, lead fracture, allergy, surgical site discomfort, infection, and disease progression.^1,9^ In our patient, particularly the latter 2 risks, subsumed in her poorly controlled diabetes, have precluded permanent implantation. Overall, however, given the success of this trial therapy in this patient, we believe that technical challenges and possible complications alone should not contraindicate thoughtful attempts at percutaneous placement.

## Disclosures

The authors have no conflicts of interest to declare.
